# Enhancing voluntary imitation through attention and motor imagery

**DOI:** 10.1007/s00221-016-4570-3

**Published:** 2016-02-18

**Authors:** Judith Bek, Ellen Poliakoff, Hannah Marshall, Sophie Trueman, Emma Gowen

**Affiliations:** School of Psychological Sciences, University of Manchester, Zochonis Building, Brunswick Street, Manchester, M13 9PL UK; Faculty of Life Sciences, University of Manchester, Manchester, UK

**Keywords:** Imitation, Action observation, Motor imagery, Attention

## Abstract

Action observation activates brain areas involved in performing the same action and has been shown to increase motor learning, with potential implications for neurorehabilitation. Recent work indicates that the effects of action observation on movement can be increased by motor imagery or by directing attention to observed actions. In voluntary imitation, activation of the motor system during action observation is already increased. We therefore explored whether imitation could be further enhanced by imagery or attention. Healthy participants observed and then immediately imitated videos of human hand movement sequences, while movement kinematics were recorded. Two blocks of trials were completed, and after the first block participants were instructed to imagine performing the observed movement (Imagery group, *N* = 18) or attend closely to the characteristics of the movement (Attention group, *N* = 15), or received no further instructions (Control group, *N* = 17). Kinematics of the imitated movements were modulated by instructions, with both Imagery and Attention groups being closer in duration, peak velocity and amplitude to the observed model compared with controls. These findings show that both attention and motor imagery can increase the accuracy of imitation and have implications for motor learning and rehabilitation. Future work is required to understand the mechanisms by which these two strategies influence imitation accuracy.

## Introduction

Perception and action are linked via an action observation network, whereby observation of another person’s movement activates brain areas involved in performing the same movement (Cross et al. 2009; Rizzolatti and Craighero 2004). Functional neuroimaging studies have revealed that action observation activates a fronto-parietal network implicated in motor execution, including ventral premotor cortex, pars opercularis of inferior frontal gyrus and inferior parietal lobe (e.g. Buccino et al. 2001; Iacoboni et al. 1999). Action observation has also been shown to increase transcranial magnetic stimulation-induced corticomotor excitability (Clark et al. 2004; Sakamoto et al. 2012). Importantly, this action observation network can be activated without the observer intending to replicate the movement, and this is seen behaviourally in visuomotor priming, also known as “automatic imitation”. In visuomotor priming studies, the observer performs a specified action while viewing another, compatible or incompatible action (e.g. Brass et al. 2001; Gowen et al. 2010; Vogt et al. 2003). Response times typically decrease when the observed action is compatible with the required response and increase when the observed action is incompatible with the response; for example, a button press response is facilitated by observing a downward finger movement but impeded by viewing an upward movement (Brass et al. 2001).

In contrast to visuomotor priming, voluntary imitation (henceforth termed *imitation)* involves the deliberate replication of an observed action, engaging the motor system through both observation and execution (Shmuelof and Zohary 2008; Small et al. 2012). Imitation is an important process in learning (Iacoboni et al. 1999) as well as having a role in social understanding and interaction (e.g. Chartrand and Bargh 1999; Meltzoff and Decety 2003). Compared with physical practice or action observation alone, imitation is associated with increased neural activations and greater effects on motor learning in healthy adults (Buccino et al. 2004; Macuga and Frey 2012; Stefan et al. 2008; Tremblay et al. 2008). Consequently, there has been increasing interest in the therapeutic potential of imitation, and recent studies have indicated positive behavioural effects of imitation training in stroke ( Buccino et al. 2006; Lee et al. 2013; Small et al. 2012), Parkinson’s disease (Buccino et al. 2011) and cerebral palsy (Buccino et al. 2012), with associated neural changes (Buccino et al. 2006).

It has previously been assumed that observed actions automatically activate the action observation network (e.g. Iacoboni et al. 1999, 2005). However, factors such as attention (Bach et al. 2007; Chong et al. 2008, 2009; Gowen et al. 2010) and intention (Badets et al. 2006; Buccino et al. 2004; Grezes et al. 1998) can influence visuomotor priming and sensorimotor activation, and recent models of imitation and visuomotor priming have incorporated top-down modulation (Gowen and Poliakoff 2012; Heyes 2011; Wang and Hamilton 2012). In the current work, we investigated whether imitation could be enhanced by manipulating instructions relating to motor imagery or attention, with a view to increasing the effectiveness of imitation in training and therapeutic settings.

Attention has been shown to improve observational learning of motor skills; for example, the addition of verbal and visual cues increased learning of a kicking action from observation of a model (Janelle et al. 2003). In a recent study, Hayes, Roberts, Elliot and Bennett (2014) examined the effects of manipulating attention during observational learning of human movement sequences depicted by a mouse cursor. Instructing participants to attend to the movement trajectory improved the accuracy with which the timing and spatial position of peak velocity were subsequently reproduced, whereas dividing attention with a concurrent tone-counting task reduced imitation accuracy.

Motor imagery (MI), or the simulation of movement in the absence of overt action (Jeannerod 1994), also activates areas of the motor system involved in action observation and execution (Anderson and Lenz 2011; Decety and Grezes 1999). As well as visual representations, MI can also involve kinaesthetic (sensorimotor) representations, whereby the sensation associated with performing the action is simulated (McAvinue and Robertson 2008; Smyth and Waller 1998). MI has been shown to enhance movement and learning in healthy adults (see review by Malouin et al. 2013), and a recent study demonstrated that visuomotor priming can be increased when action observation is combined with MI (Eaves et al. 2014). Participants viewed an image of a to-be-executed rhythmical action (e.g. face-washing), which was followed by a video of a different, distractor action (e.g. painting). When participants were asked to imagine performing the to-be-executed action to the rhythm of the distractor action, the cycle time of the subsequently executed action was more strongly biased towards that of the observed action than with observation alone. In addition, neuroimaging studies have revealed stronger activations across motor areas during concurrent observation and MI than during action observation alone, including regions of premotor cortex, inferior parietal cortex and insula (Macuga and Frey 2012; Nedelko et al. 2012; Villiger et al. 2013). Increased desynchronisation in sensorimotor areas during action observation combined with MI has also been found using electroencephalography (Berends et al. 2013). Although MI and AO could potentially compete for the same representational resources, neural and behavioural evidence suggests that, as long as the imagined action complements rather than conflicts with the observed action, combined AO and MI has a facilitatory effect on movement (Vogt et al. 2013). Vogt et al. (2013) have conceptualised AO and MI as externally guided and internally guided forms of motor simulation, respectively.

While it has been demonstrated that MI and attention can increase visuomotor priming and enhance learning from observed actions, effects on imitation require further investigation. During imitation, sensorimotor representations may already be activated more strongly, since the observer is preparing to perform the observed action (Badets et al. 2006; Buccino et al. 2004; Grezes et al. 1998). Therefore, manipulations designed to increase attention or promote MI may have less of an effect on imitation than in visuomotor priming. That is, when an action is being observed with the intention to imitate, attention and MI might confer no additional benefit. The present study addressed this question by exploring whether imitation of human movement could be enhanced by combining observation with MI, or by increasing attention to characteristics of the observed stimulus.

In the first part of the experiment, participants observed and then immediately imitated a human hand moving sequentially between target locations. An atypical, elevated trajectory was used for the movement so that similarity between the participant’s movements and those of the model was likely to reflect imitation, rather than coincidental similarity (see Hayes et al. 2014). In the second part of the experiment, participants were given instructions to either promote the use of MI or increase attention to the observed movements, while a control group received no additional instructions. MI instructions were designed to prompt participants to engage in kinaesthetic imagery, as this has been found to activate the motor system more strongly than visual imagery (Anderson and Lenz 2011; Guillot et al. 2009; Voisin et al. 2011), as well as being consistent with previous studies (Eaves et al. 2012, 2014). Kinematics of the imitated actions were analysed, examining the effects of attention and MI on duration, peak velocity and amplitude of imitated movements, as well as accuracy in relation to the model’s kinematics and variability of the movement.

It was expected that instructing participants to engage in MI, or to attend to the characteristics of the observed action, would increase the accuracy of imitation. No specific prediction was made concerning the relative effects of MI and attention instructions, since these have not been directly compared in previous studies. We also explored whether the effects of MI and attention instructions might be mediated by the presence of movement goals. According to the goal-directed theory of imitation (Bekkering et al. 2000), observed actions directed towards a visible target are primarily coded in terms of the goal or target of the action, rather than the characteristics of the movement itself. In the absence of goals, greater weighting is placed on encoding the movements, resulting in more accurate imitation of kinematics (Wild et al. 2010). Consequently, it is possible that attention to the movement and spontaneous use of MI might be greater for goal-less than goal-directed actions. Prompting participants to attend to the characteristics of the movement, or to engage in MI, might therefore have a greater influence on imitation of goal-directed actions by altering the weighting of movement characteristics relative to goals. Alternatively, increasing attention or MI might improve imitation accuracy for both types of action.

## Method

### Participants

Participants were undergraduate students from the University of Manchester who received course credit for their participation. Participants were randomly allocated to one of three groups (Imagery, Attention or Control). The Imagery group (*N* = 18, 5 males) had a mean age of 19.4 ± .98 years, the Attention group (*N* = 15, 2 males) had a mean age of 19.9 ± 1.4 years, and the Control group (*N* = 17, 1 male) had a mean age of 19.8 ± 1.7 years. The groups did not differ significantly in age [*F*(2,49) = .57; NS] or sex [*χ*^2^(2) = 3.23; NS].

All participants were right-handed and had normal or corrected-to-normal vision and no history of neurological illness or injury. The study was approved by the University of Manchester Research Ethics Committee.

### Experimental set-up

The stimuli and protocol were based closely on previous work by the authors (Wild et al. 2010, 2012). The participant was seated at a desk with their right hand occluded by a box measuring 65 cm × 45 cm × 20 cm. Stimuli were displayed using Presentation software (Neurobehavioural Systems) and projected at life size onto a 100 cm × 75 cm screen at a distance of 120 cm from the participant. Kinematic data were collected using a Polhemus Liberty Motion Tracker with Motion Monitor software. Movement coordinates in the *x*, *y* and *z* axes were recorded at a sampling rate of 120 Hz via a sensor attached to the intermediate phalanx of the index finger of the right hand.

### Stimuli

**Fig. 1:**
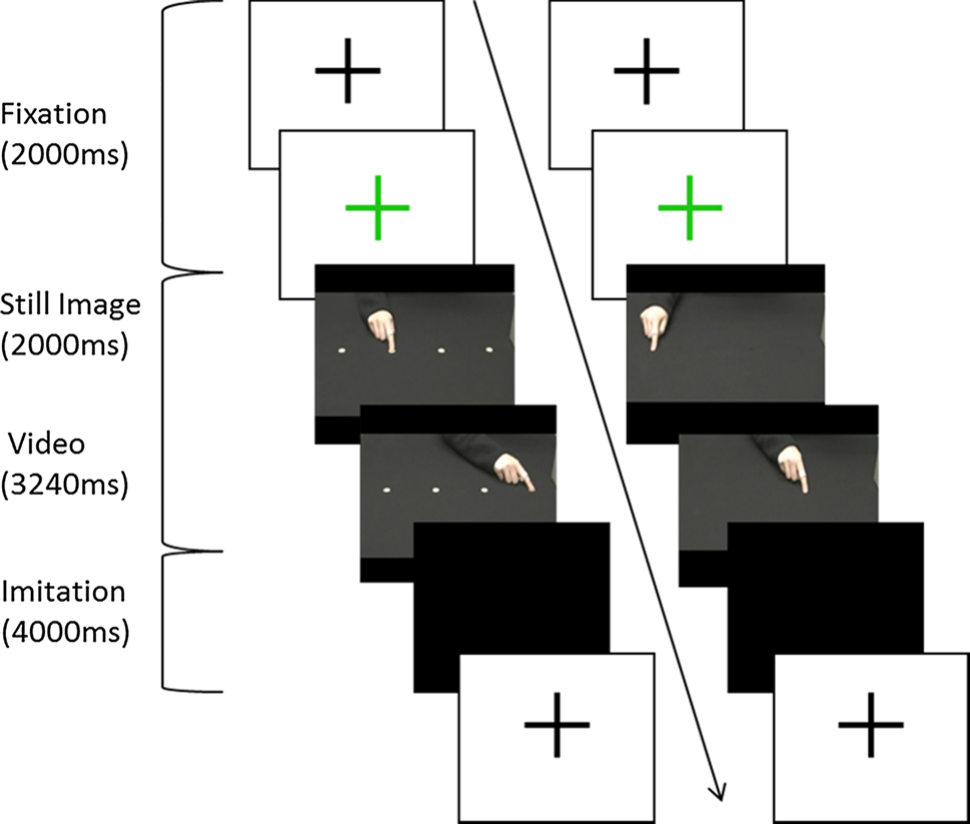
Time lapse diagram of the trial sequence, showing a target (T) trial (left) and non-target (NT) trial (right). Figure adapted from Wild et al. (2012).

Stimuli were video clips of finger movement sequences made by a human hand, visible to just beyond the wrist (see Fig. 1). Sequences consisted of two movements between three out of four possible positions spaced 15 cm apart in the horizontal plane. The video-recorded movements were paced using a metronome, and the kinematics of the model’s movements were measured during recording. For non-target (NT) trials, the hand was displayed against a dark grey background with no visible targets. The stimuli used for target (T) trials were identical except that the four possible target locations were marked by small light grey circles, each measuring 19 mm in diameter.

As in our previous work (Wild et al. 2010, 2012), sequences that included a movement between the second and fourth locations were selected for analysis. This was one of the larger movements, and the second location was not close to the periphery of the visual area, so the edges of the desk or screen could not be used as reference points for the location.

### Procedure

As illustrated in Fig. 1, each trial began with a fixation cross (55 × 55 mm) that flashed from black to green (2000 ms). This was followed by a still image indicating the starting position for the sequence, which was displayed for 2000 ms while the participant placed their right index finger in the position indicated. The stimulus video was then displayed for 3240 ms, which showed the finger moving from the initial position to a second and then a third position. Following stimulus presentation, a blank screen was displayed for 4000 ms, during which participants were required to imitate the observed movement sequence using their right hand. Participants were instructed to “watch the video clip carefully and copy what you see”.

Two blocks of 30 trials were completed with a short break halfway through each block. Each block consisted of 15 trials with visual targets (T) and 15 trials without visual targets (NT), 10 of each containing the to-be-analysed 4–2 movement. Trials containing the 4–2 movement (4–2–1, 4–2–3) were interspersed with other sequences^1^ to reduce predictability. Trial order was randomised within blocks and between participants.

After the first block of 30 trials, the two experimental groups were given written instructions designed either to prompt the use of MI (Imagery group) or to increase attention to the stimuli (Attention group). Participants in the Imagery group received the following instruction: “Imagine what it feels like to make the movements yourself. As you watch the demonstrator’s hand move from one place to another, imagine what your arm, hand and finger would feel like to copy the movements”. The participants in the Attention group were given the following instruction: “Pay close attention to the specific movement made. For example, look at how fast the action is carried out, and the size of the movement, so how high the hand is lifted, and exactly where the movement occurs from and to”. The Control group received no further instructions but instead rested for 2 min between blocks.

### Data analysis

Kinematic data from correctly executed 4–2 movements were analysed using MATLAB. Errors or missing data led to the exclusion of 1 trial from the Imagery group, 3 trials from the Attention group and 9 trials from the Control group. Onset and offset times for each movement were, respectively, determined when velocity rose above or fell below 10 % of the peak velocity for 6 consecutive samples (48 ms). For each participant, mean movement duration, peak velocity, time taken to reach peak velocity, horizontal amplitude and vertical amplitude were then calculated for T and NT trials.

For each movement parameter, all trials were first screened for outliers at the individual level (based on procedure recommended by Van Selst and Jolicoeur 1994) for each stimulus type (T/NT) and each time point (pre-/post-instruction).^2^ For each group, means for each stimulus type (T/NT) and time point (pre-/post-instruction) were then calculated and outliers were identified and excluded at the participant level. This resulted in the exclusion of data from two participants in the Attention group for peak velocity, 2 participants for time to peak velocity (1 Attention and 1 Control), 4 participants for horizontal amplitude (2 Control, 1 Attention and 1 Imagery) and 2 participants for vertical amplitude (1 Attention and 1 Control).

Initial ANOVAs examined the effects of stimulus type (T/NT), instruction (Imagery/Attention/Control) and time point (pre-/post-instruction) on each movement parameter. ANCOVAs were used to examine the effect of instruction (Imagery/Attention/Control) on each parameter, to control for any differences between groups in pre-instruction performance and to address within-group variance. Levene’s test for homogeneity of variance was significant for movement duration (*p* < .05), so duration data were log-transformed prior to further analysis.

To assess imitation accuracy in relation to the observed (model) movements, absolute error was calculated for each movement parameter by subtracting the participant’s value on each trial from the model value (recorded during filming of the stimulus videos) and taking the mean of absolute (unsigned) differences between the participant and model. Variable error was also analysed; this was calculated for each movement parameter as the mean unsigned difference between the value for each trial and the participant’s mean (Schmidt and Lee 2011). For absolute and variable error, paired *t* tests were used to compare the first versus second block for each group. To correct for multiple comparisons, a conservative significance level of *p* < .017 was adopted.

## Results

**Fig. 2:**
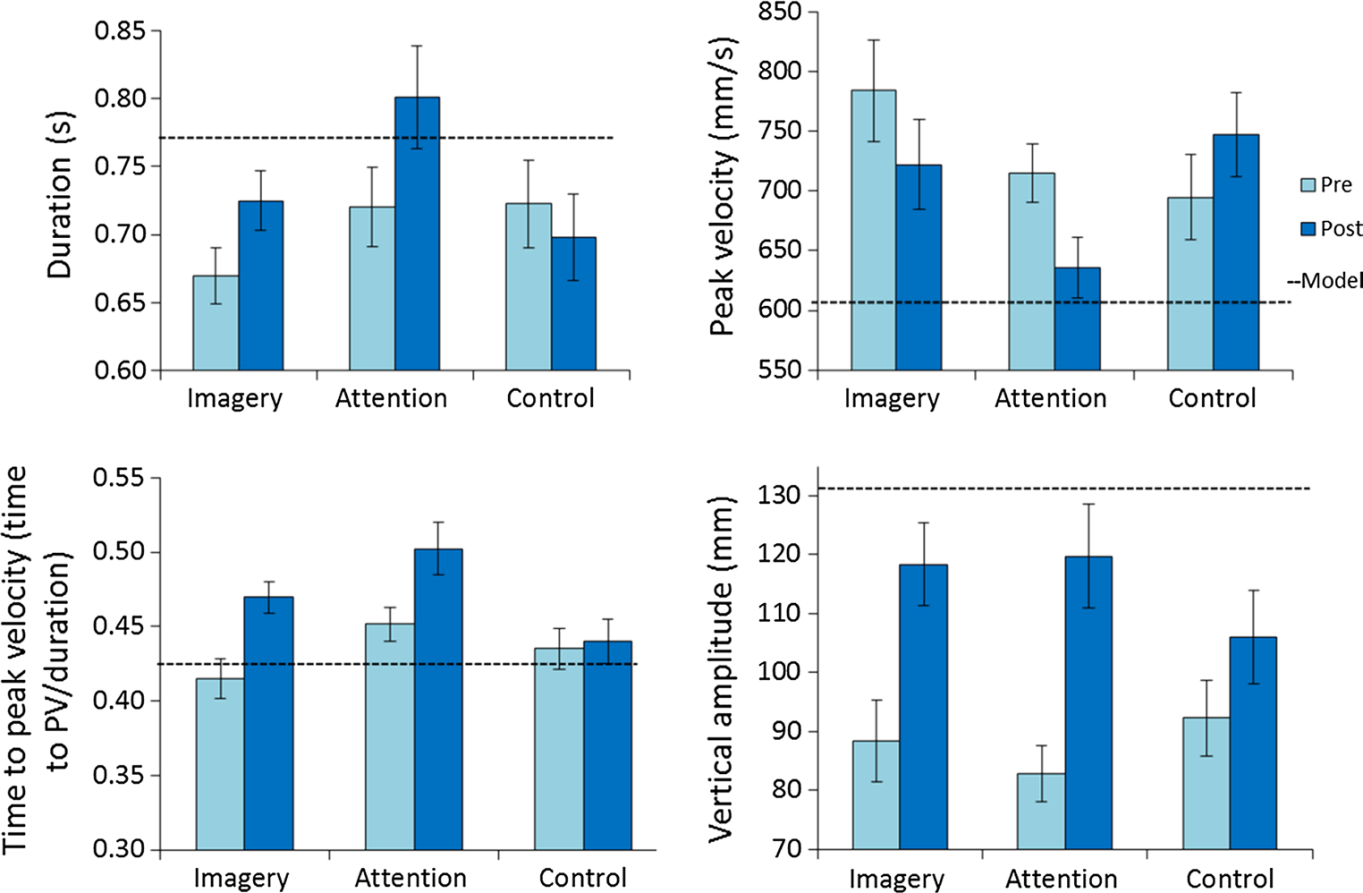
Movement parameters pre- and post-instruction in Imagery, Attention and Control (no instruction) groups. The dashed line shows the value of the observed action. *Error bars* represent ±1SEM

Results for each movement parameter are illustrated in Fig. 2. The black dashed line represents the model’s kinematics, recorded during filming of stimulus videos. Absolute error scores (model—participant) are presented in Table 1, and variable error is shown in Table 2. Initial ANOVAs (stimulus type × instruction × time point) revealed a significant main effect of target (target/no-target) on peak velocity [*F*(1,46) = 9.38; *p* = .004; *η*^2^*p* = .17], which was higher in target (T) than in non-target (NT) trials. However, there were no other main effects of target and no interactions with time (pre/post) or instruction (Imagery/Attention/Control). T and NT trials were therefore collapsed for subsequent analysis.

**Table 1:** Absolute error between participant and model values: *M* ± SD and *t* values for each instruction group pre- and post-instructions

	Imagery				Attention				Control			
	Pre	Post	*t*	*p*	Pre	Post	*t*	*p*	Pre	Post	*t*	*p*
Duration (s)	.13 ± .05	.11 ± .05	−.95	.13	.12 ± .06	.13 ± .08	.65	.36	.15 ± .05	.14 ± .07	.87	.40
Peak velocity (mm/s)	194.8 ± 131.3	149.8 ± 109.9	2.05	.057	123.1 ± 48.2	99.8 ± 39.5	1.59	.14	155.7 ± 62.2	161.5 ± 94.8	−.37	.72
Time to peak velocity (time/duration)	.07 ± .02	.07 ± .02	.34	.74	.07 ± .02	.10 ± .05	−3.08	.008*	.08 ± .02	.08 ± .03	1.00	.33
Vertical amplitude (mm)	47.4 ± 20.8	30.8 ± 14.5	3.72	.002*	48.8 ± 17.1	33.0 ± 17.7	3.10	.008*	41.8 ± 21.6	38.1 ± 18.8	1.32	.21
Horizontal amplitude (mm)	43.1 ± 21.3	47.6 ± 25.5	−.20	.84	33.1 ± 17.0	37.6 ± 20.2	−1.65	.12	44.5 ± 32.9	46.3 ± 37.0	−.62	.55

* *p* < .017

**Table 2:** Variable error: *M* ± SD and *t* values for each instruction group pre- and post-instructions

	Imagery				Attention				Control			
	Pre	Post	*t*	*p*	Pre	Post	*t*	*p*	Pre	Post	*t*	*p*
Duration	.07 ± .02	.08 ± .02	−2.16	.045	.08 ± .02	.10 ± .03	−2.82	.014^*^	.11 ± .04	.08 ± .02	3.73	.002*
Peak velocity	85.4 ± 22.1	83.7 ± 23.7	.21	.83	106.4 ± 34.5	83.1 ± 19.0	2.49	.027	106.5 ± 32.5	92.2 ± 33.0	1.80	.09
Time to peak velocity	.07 ± .02	.07 ± .02	−.19	.85	.08 ± .02	.09 ± .03	−1.33	.20	.09 ± .02	.07 ± .02	2.27	.038
Vertical amplitude	14.3 ± 7.0	18.7 ± 4.9	−2.66	.017	14.0 ± 3.6	21.3 ± 7.1	−4.41	.001*	14.7 ± 4.4	17.5 ± 7.2	−2.43	.028
Horizontal amplitude	35.7 ± 36.0	40.2 ± 50.6	−.37	.71	29.7 ± 16.7	39.5 ± 40.8	−1.18	.26	47.0 ± 44.3	41.9 ± 45.5	.42	.68

* *p* < .017

ANCOVA revealed a significant effect of instruction on movement duration [*F*(2, 49) = 14.3; *p* < .001; *η*^*2*^*p* = .38], which was longer (closer to the model) in the Imagery group (adjusted *M* = .76; *p* = .003) and the Attention group (adjusted *M* = .80; *p* < .001) relative to the Control group (adjusted *M* = .68), but did not differ significantly between Attention and Imagery groups. Following instructions, absolute error did not change significantly in any of the three groups. There was a significant increase in variability of duration in the Attention group, while variable error decreased in the Control group.

There was a significant effect of instruction on peak velocity [*F*(2, 49) = 10.08; *p* < .001; *η*^*2*^*p* = .31], which was significantly lower (closer to the model) in the Imagery group (adjusted *M* = 683.20; *p* = .006) and the Attention group (adjusted *M* = 649.67; *p* < .001) compared with controls (adjusted *M* = 777.16). There was no significant difference between Attention and Imagery groups. Absolute and variable error of peak velocity did not differ significantly between blocks in any of the groups.

Time to peak velocity also showed a significant effect of instruction [*F*(2, 48) = 32.7; *p* = .008; *η*^*2*^*p* = .19], with the Control group exhibiting significantly shorter time to peak velocity, which was closer to the model (adjusted *M* = .44) than both the Imagery group (adjusted *M* = .49; *p* = .017) and the Attention group (adjusted *M* = .48; *p* = .037), but there was no significant difference between Attention and Imagery groups. Following instructions, the Attention group showed a significant increase in absolute error for time to peak velocity. Absolute error did not change significantly in the Imagery group or the Control group. Variability of time to peak velocity did not change significantly in any group.

There was a significant effect of instruction on vertical amplitude [*F*(2, 48) = 3.89; *p* = .028; *η*^*2*^*p* = .15], reflecting marginally significantly larger amplitude (closer to that of the model) in the Imagery (adjusted *M* = 118.10; *p* = 0.058) and Attention (adjusted *M* = 119.31; *p* = .062) groups than in the Control group (adjusted *M* = 98.49). There was no significant difference between Imagery and Attention groups. There was a significant decrease in absolute error of vertical amplitude in the Imagery group and the Attention group post-instructions, while the Control group did not show any significant change in accuracy. Variability of vertical amplitude increased significantly between blocks in the Attention group and marginally in the Imagery group.

No significant effect of instruction was found for horizontal amplitude [*F*(2, 47) = .85; NS]. Absolute and variable error did not change significantly between the two blocks in any of the groups.

## Discussion

The present study examined how instructions designed to increase motor imagery or attention affected the voluntary imitation of human hand movement sequences. Kinematics of hand movements were altered by both types of instruction, with no significant differences between groups instructed to attend closely to or imagine themselves performing the observed movements. Movement duration and time taken to reach peak velocity were longer, vertical amplitude was greater, and peak velocity was lower in participants given MI or attention instructions compared with a control group. With the exception of time to peak velocity, these differences reflected movements that were more similar to the observed model following instruction. As the observed movements involved an elevated trajectory that was unnecessary to achieve the end point of the movement (the target position could be reached by a more direct movement of lower amplitude), the increase in vertical amplitude with MI and attention instructions in particular indicates that imitation was enhanced. No differences between groups were found for horizontal amplitude. This is perhaps to be expected, since simply attending to the end point of the movement would have been sufficient to allow replication of the horizontal amplitude, with attention and MI instructions not contributing further to accuracy.

Further analysis comparing kinematics with those of the model’s movement revealed increased accuracy (decreased absolute error) in vertical amplitude with both attention and MI instructions. However, accuracy of time to reach peak velocity decreased in the Attention group. This suggests that the attention instructions may have led participants to attend more closely to the elevation of the observed movements rather than duration and velocity. Hayes et al. (2014) also found that the effects of attention instructions differed for different parameters of movement, such that directing attention to the movement trajectory increased accuracy of peak velocity but decreased accuracy of movement duration. Indeed, the effects of attentional manipulations may depend upon which aspects of movement are emphasised in the task instructions. In the present study, participants in the Attention group were asked to attend to both the speed and amplitude, but perhaps with greater emphasis on amplitude. Although time to peak velocity was closer to the model in the Control group than in the experimental groups, accuracy did not change between blocks, indicating that practice effects were not responsible for this group difference. Indeed, accuracy did not change between blocks for any of the movement parameters in the Control group, indicating that the effects of MI and attention instructions were not due to practice alone.

Although some increases in accuracy were found with attention and MI instructions, variability of vertical amplitude increased in both groups and variability of duration increased in the Attention group. It has been suggested that variability is more sensitive to the effects of learning (Schmidt and Lee 2011), and the increase in variability might reflect the learning process as participants attempted to apply different strategies to the imitation task. Alternatively, the increased variability might reflect fatigue or motivational factors, such that accuracy increased initially but decreased in later trials as participants applied the attention or MI instructions less consistently. In contrast, the Control group showed decreased variability of duration, which is likely to reflect an effect of practice.

Our results add to existing evidence that the effects of action observation can be enhanced by MI (Eaves et al. 2014) and attention (Bach et al. 2007; Chong et al. 2009; Gowen et al. 2010; Hayes et al. 2014). The present findings are also consistent with recent models emphasising modulation of visuomotor priming and imitation (Gowen and Poliakoff 2012; Heyes 2011; Wang and Hamilton 2012). In the most relevant previous study, Hayes et al. (2014) found that attention to the trajectory of observed movements during a training period increased the accuracy of subsequently executed hand movements. In the present study, however, participants were required to imitate hand movements directly after observation; our findings thus demonstrate that attention and MI can also influence kinematics during immediate imitation of human movement.

Demonstrating effects of attention and MI on imitation is particularly important as it has been shown that the instruction to imitate already increases activation of sensorimotor representations, since the observer is preparing to perform the observed action. For example, intention to imitate during action observation has been shown to improve the timing of subsequent actions compared to verbally describing the timing characteristics (Badets et al. 2006). Since both preparing to imitate an action and describing the action require attention to the movement, this finding suggests that intention to imitate may have increased sensorimotor activation or simulation (see also, Buccino et al. 2004; Grezes et al. 1998), rather than simply drawing attention to the stimulus. However, our findings demonstrate that it is possible to increase imitation accuracy further through both MI and attention manipulations. Facilitating imitation has implications for the use of imitation-based training and therapies in conditions where the ability to imitate may be compromised, such as in stroke and Parkinson’s disease (e.g. Bonivento et al. 2013; Desmarais et al. 2006; Hoeren et al. 2014; Leiguarda et al. 1997). Enhancing imitation might also be beneficial in populations where difficulties in imitating may impact on social understanding and interaction, such as in autistic spectrum conditions (Gowen 2012; Spengler et al. 2010; Wild et al. 2012).

It was anticipated that instructions might enhance imitation to a greater extent for goal-directed actions, such that processing of kinematics relative to movement goals (targets) would be enhanced with increased attention to the characteristics of the movement or increased MI. However, other than for peak velocity, instructions did not differentially affect imitation of movements with and without visual targets. This may, in part, be explained by reference to the elevated hand movements in the observed stimuli. Since the elevated motion was not necessary to achieve the end point of the action, it is possible that attention was drawn more strongly to the unusual nature of the movement than to the targets. Indeed, using the same stimuli, Wild et al. (2012) found that even without specific instructions participants imitated the kinematics of both goal-directed and goal-less actions during elevated trials. Moreover, the targets in the present study were quite subtle (small grey-coloured circles) and may not have been sufficiently salient to prompt goal-based coding when combined with the elevated motion. Further research could explore the effects of instructions on imitation of other types of goal-directed movement.

Although MI and attention instructions both enhanced imitation accuracy, the mechanisms underlying these effects are unclear. One possible explanation is that both sets of instructions enhanced visual processing of the observed stimuli, such that attention was heightened in participants attempting to engage in MI as well as in those instructed to attend more closely. Alternatively, both manipulations may have increased the use of MI. MI may involve visual or kinaesthetic (sensorimotor) processes to different degrees (McAvinue and Robertson 2008; Smyth and Waller 1998), with kinaesthetic imagery being associated with stronger sensorimotor activations, whereas visual imagery appears to recruit occipital regions involved in visual processing (Anderson and Lenz 2011; Guillot et al. 2009; Voisin et al. 2011). In the present study, as well as that of Eaves et al. (2014), participants were instructed to engage in first-person, kinaesthetic imagery. In contrast, asking participants to attend to the characteristics of the movement may evoke a more visual form of imagery. A final possibility is that the different instructions resulted in similar effects on imitation but via different mechanisms, such that attention instructions enhanced visual processing, while MI instructions increased simulation.

One issue in determining the contributions of MI and attention to imitation is the difficulty in measuring MI. It is not known to what extent MI is spontaneously engaged during action observation; similarly, it is not clear how the extent or vividness of MI is affected by instructions such as those used in the present study. In this respect, it might be informative to analyse imitation performance in relation to measures of MI ability, although behavioural measures of imagery ability do not provide information on the use of MI during a specific task. Alternatively, future research could explore MI and attention online during imitation, using methods such as neuroimaging to examine visual and sensorimotor activations (e.g. Guillot et al. 2009) or eye tracking (e.g. Heremans et al. 2008, 2011; Wild et al. 2012).

The design and approach of the present study could be developed in a number of ways in future work. First, in keeping with previous studies (Eaves et al. 2012, 2014), we presented actions from a third-person (external) perspective. It is possible that stronger effects of MI instructions would be found for stimuli viewed from the first-person perspective, as observed and imagined action would be more closely matched. Second, the timing of MI might influence its efficacy; for example, employing MI between observation and execution could help to consolidate motor representations. Third, our stimuli were anatomically matched to the observer (i.e. participants observed a right hand and responded with their right hand), whereas evidence suggests that the action observation network responds preferentially to mirror-image stimuli, such as observing a left hand and responding with the right (Chiavarino et al. 2007; Shmuelof and Zohary 2008). Therefore, congruency with the observer’s posture or effector warrants investigation. Nevertheless, in previous work we found evidence of imitation for anatomically matched stimuli (Wild et al. 2010, 2012), and it is encouraging that the present results show that imitation of these stimuli can be enhanced by MI and attention instructions, although stronger effects of MI instructions might be found for mirrored stimuli.

In conclusion, instructing participants to attend closely to hand movement sequences, or to imagine themselves performing the observed actions, altered imitation of kinematics compared with a control group. This resulted in movements that were more similar to those of the observed model, demonstrating for the first time that both MI and attention can influence the immediate imitation of a simple two-movement sequence. More broadly, the present results add to existing evidence that the effects of observed actions on the motor system can be influenced by top-down factors, which has implications for imitation-based therapies.

## Footnotes

1 Filler sequences were: 123, 134, 213, 214, 312, 341 and 413.

2 Across all groups, a total of 1.1 % of trials were excluded for duration, 1.3 % for peak velocity, 0.9 % for time to peak velocity, 1.9 % for horizontal amplitude and 1.1 % for vertical amplitude.
